# Are childcare settings’ food menus fit for purpose? A qualitative analysis in England

**DOI:** 10.1093/heapro/daaf179

**Published:** 2025-10-30

**Authors:** Emily Warren, Lorraine Williams, Josephine Exley, Paul Boadu, Bob Erens, Dayna Brackley, Rosie Osborne, Cécile Knai

**Affiliations:** Faculty of Public Health and Policy, London School of Hygiene & Tropical Medicine, 15-17 Tavistock Place, London WC1H 9SH, United Kingdom; The Food Policy Impact Lab, Faculty of Public Health and Policy, London School of Hygiene & Tropical Medicine, 15-17 Tavistock Place, London WC1H 9SH, United Kingdom; Faculty of Public Health and Policy, London School of Hygiene & Tropical Medicine, 15-17 Tavistock Place, London WC1H 9SH, United Kingdom; Faculty of Public Health and Policy, London School of Hygiene & Tropical Medicine, 15-17 Tavistock Place, London WC1H 9SH, United Kingdom; Faculty of Public Health and Policy, London School of Hygiene & Tropical Medicine, 15-17 Tavistock Place, London WC1H 9SH, United Kingdom; Faculty of Public Health and Policy, London School of Hygiene & Tropical Medicine, 15-17 Tavistock Place, London WC1H 9SH, United Kingdom; Bremner & Co, Nest 58 floor, Victoria Embankment, London EC4Y ODS, United Kingdom; Bremner & Co, Nest 58 floor, Victoria Embankment, London EC4Y ODS, United Kingdom; Faculty of Public Health and Policy, London School of Hygiene & Tropical Medicine, 15-17 Tavistock Place, London WC1H 9SH, United Kingdom; The Food Policy Impact Lab, Faculty of Public Health and Policy, London School of Hygiene & Tropical Medicine, 15-17 Tavistock Place, London WC1H 9SH, United Kingdom

## Abstract

Childcare settings have a central role in feeding pre-school-aged infants and children. One of the ways in which childcare settings plan nutritious, balanced, and varied meals and snacks for preschool-aged infants and children (0–5 years) is through the use of a menu. Nevertheless, international studies indicate an overwhelming heterogeneity in uptake of menus, as well as use and format, with variable details of food and drinks provided. Thus, in the context of a nationally representative survey on food provision in early years settings in England, we invited respondents to upload sample menus. Of the 322 settings that completed the survey, 56 submitted menus (17.4%). Five were excluded because the attachment was either not a menu or was illegible. Data contained in the 51 readable menus was extracted into an Excel spreadsheet designed deductively from available guidance on menus and inductively based on patterns emerging from the menus themselves. The menus demonstrated great variations in depth of information, completeness and clarity. Breakfasts, snacks, and beverages were often excluded from menus or the information about them was unclear. Menus also sometimes contained dishes with names that were unclear. Early years settings are expected to promote healthy eating, but their ability to do so is shaped by wider structural factors. Thus while childcare settings can play a crucial role in the health promotion of young children during a time of vital development, the wider policy context and challenges faced by childcare settings and families must be addressed.

Contribution to Health Promotion

- This study is a qualitative exploration of 51 childcare setting menus voluntarily provided by childcare settings themselves in the context of a national survey of food in childcare settings in England;
- We found that the depth of information and quality of menus varied widely, with the majority assessed as not providing enough detail to inform parents or carers, or allow for meaningful assessment of the nutritional content of meals;
- This study emphasizes the need to contextualize, and respond to, these findings in the wider challenges faced by childcare settings in England, as explained by the Framework for Childhood Health Promotion;
- Childcare settings have a considerable potential to promote health more effectively, e.g. by enabling the provision of healthy foods via high quality menus, but only if the wider policy context and challenges faced by childcare settings and families are addressed.

## INTRODUCTION

Childcare settings can be sites of health promotion for young children during a time of vital physical, mental and social development. Though this paper uses the term ‘childcare setting’, the terminology used across the world is varied, such as kindergarten (Fjæra *et al*. 2025), early learning services (Hall *et al*. 2025), preschools (Stojisavljevic *et al*. 2025), childcare settings (Elford *et al*. 2022), long day care centres, early childhood education and care centres (Searle *et al*. 2025) and nurseries (González-García *et al*. 2021). In England, various terms are also used, including children’s centres, early years settings, and childminders (Goldsborough *et al*. 2016).

Childcare settings have a central role in feeding pre-school-aged infants and children. In England, a child attending a typical full-day childcare setting will eat at least two if not three meals and two snacks per day there, representing approximately 90% of their calories, making these settings vital for health promotion (Mucavele *et al*. 2020). This is all the more important as early childhood is a particularly vulnerable life stage. The extent to which healthy diets are available in early life can affect the current health and future development and opportunity of an individual child, and can widen inequities (Mistry *et al*. 2012, Pearce *et al*. 2019).

One of the ways in which childcare settings plan nutritious, balanced, and varied meals and snacks for preschool-aged infants and children (0–5 years) is through the use of a menu (Department for Education 2025). A menu has a range of important function for childcare settings, such as facilitating communication with parents and carers of prospective and currently-enrolled children (e.g. regarding allergens and dietary restrictions), and providing evidence of compliance with national standards and frameworks. Menus also help settings balance nutrients and meals across their weekly, monthly, or season cycle, and can aid in the efficient planning and purchasing of ingredients.

In recent years, there has been a surge of research on childcare setting menus, especially in Australia and New Zealand. Studies in Australia have found that better compliance to sector dietary guidelines is associated with shorter menu cycles and the higher take-up of training opportunities (Grady *et al*. 2020). Evidence also suggests that, menu quality improves where free menu planning support (whether in-person or online) is available (Elford *et al*. 2022). Moreover, the requirement to provide written menus is not sufficient to demonstrate that food provided by a setting is either nutritious or adequate in quantity (Searle *et al*. 2025). In New Zealand, studies indicate the receipt of an externally awarded health award to childcare settings being statistically associated with high menu scores on quantity, variety and quality (Gerritsen *et al*. 2017); and that menus most often did not meet quality standards in terms of meeting national guidelines as well as other quality criteria (McKelvie-Sebileau *et al*. 2022, Hall *et al*. 2025). Studies from other countries shows benefits of using menus, including using menu planning, to reduce food waste in Finland (Silvennoinen *et al*. 2025) and reducing the water and carbon footprint of nursery food in Spain (González-García *et al*. 2021); as well as challenges of implementing meal planning and menus in Norway (Fjæra *et al*. 2025).

In England, existing studies are limited and often focused at local level. Buttivant and Knai (2012)conducted a qualitative case study of food provision in childcare in the city of Southampton, England; they found that few settings had a food plan or menu plans and that the lack of authoritative guidance led to confusion. Goldsborough *et al* (2016) studied healthy eating in eight childminder settings in Rotherham, Yorkshire in England and found that following menu plans was reported to be difficult (Goldsborough *et al*. 2016). Finally, Brackley *et al* (2024) conducted a case study of food provision in childcare across Yorkshire, including menu analysis of 20 settings: menus were found to be heterogeneous, with variable details of food provided, and not regularly updated (Brackley *et al*. 2024). Research in England with parents of children in childcare settings also indicated that menus are a key tool that parents use to assess whether their child is eating a sufficient variety of foods, whether they are served foods that align with their cultural preferences, and help address concerns, such as fussy eating (Williams *et al*. 2022). This echoes a systematic review of dietary behaviour policies in guidelines in childcare settings which identified parents engagement strategies including asking them to make suggestions to the development of a healthy childcare setting menu (Jackson *et al*. 2021).

Many children in the UK consume excess calories, saturated fat, sugar and salt (Roberts *et al*. 2018) and insufficient fruits, vegetables and fibre (Albani *et al*. 2017). The latest National Diet and Nutrition survey (2019–23) indicates that children aged 18 months to 3 years were drinking on average 33 ml of soft drinks a day, with only 27% meeting the recommendation for intake of free sugars (5% or less of energy), and only 22% meeting the recommended fibre intake (UK Government 2025a, b).

In England, all settings must comply with the Early Years Foundation Stage (EYFS) framework provided by the Office for Standards in Education, Children’s Services and Skills (Ofsted). This includes a welfare requirement stating that the food and drinks provided must be ‘healthy, balanced, and nutritious’ (Ofsted 2015, Ofsted 2019). Two separate guidance documents were developed to support this aim. The first is the ‘Eat Better, Start Better’ food and drink guidelines for childcare settings, developed by the School Food Trust (Action for Children 2017). The second set are Example Menus for Childcare Settings (HM Government 2017a, b) developed by the Government. Though neither document provided guidance on what information childcare menus should contain, the UK Department for Education (DfE) has now published new guidance on early years nutrition with information on menu planning for nurseries: specifically it provides information on taking into consideration nutritional value (nutrients, portion sizes, things to avoid), dietary requirements, variation and balance, planning menus lasting at least one week, and engaging with parents and carers about the menus (Department for Education 2025).

To our knowledge, there is still limited research in England and the UK on childcare menus, with existing studies focusing on a city or region, and none providing national insight. This exploratory qualitative analysis of childcare menus across England is, to our knowledge, the first and largest of its kind in reporting on 51 menus voluntarily provided by childcare settings themselves in the context of a national survey of food in childcare settings in England (Warren *et al*. 2024). This paper seeks to describe the depth, completeness, and clarity of information provided in menus.

### Theoretical underpinning

This study is underpinned by the Framework for Childhood Health Promotion (FCHP) which sets out the manifold influences on children’s health and how they interconnect (Mistry *et al*. 2012). According to the framework, the public and private sectors develop and enact policies and programmes intended to enhance the capacities of families and communities. Community resources include institutional resources (such as menus). These resources and capacities enable families and communities to develop the foundations of health for children to thrive. These foundations include responsive caregiving, safe and secure environments, good nutrition, and health promoting behaviours. These foundations shape the biological mechanisms that affect children throughout the life-course and their own pre-conception health, and into future generations. The effects of this chain are described as being moderated, or altered in intensity, by two key contextual influences: settings including childcare settings as well as the broad determinants of health. Settings and the determinants of health can work to promote health more effectively, e.g. by enabling settings to plan for and provide healthy foods via high quality menus.

## METHODS

### Data collection

In the context of a nationally representative cross-sectional online survey of childcare settings providing care on non-domestic premises (nurseries) on food provision and practices in England between October and November 2021, we provided an opportunity for respondents to upload their example menus. This was not a mandatory section. The survey was sent to 2860 nurseries via email, with up to three reminder emails sent to those who did not respond. Childminders, or other care providers working on domestic premises were excluded because there was not a reliable database we could use to develop a representative sample. The detailed methods of the survey itself are published elsewhere (Warren *et al*. 2024).

### Design of data extraction sheet

In order to build an appropriate data extraction sheet, all menus were read as they were submitted via the survey, and similarities and differences in each were noted. For example, on first reading, we noted large variations in the amount and types of adjectives used to describe food in submitted menus. Based on these inductively-derived observations, relevant columns were added to the data extraction sheet in an excel worksheet. Other themes for extraction were derived deductively based on the guidance provided in relevant childcare settings guidance. Examples of deductively derived themes include whether every snack and meal is listed and whether there is evidence to indicate the menu changing at least twice a year.

To both quantify and describe the findings, the data extraction sheet contained many question pairs in which the first column could be answerable with a simple ‘yes’, ‘some/somewhat’, ‘no’, or ‘non-applicable’, and the second corresponding column contained qualitative information to help us assess the nuance within the menu. For example, one column was labelled, ‘Is information about beverages on the menu?’ and the following column was labelled ‘what information about beverages is given?’

After preliminary development, the data extraction sheet was piloted by three authors, and additional inductive codes were suggested and agreed (see Supplement S1 for a list of all of the column titles in the data extraction sheet). Finally, relevant survey data from each setting that submitted a menu was added to the data extraction sheet in order to assess both alignment between the survey and the menu, as well as information relating to whether settings reported knowledge or use of key guidelines.

### Data extraction

EW extracted all information from the menus into the piloted data extraction form. To ensure high-quality data extraction, 14 menus were dual-extracted by another author (L.W.). In the event of differences in assessment, the menus were revisited and discussed.

### Data analysis

Menus were analysed according to depth, completeness and clarity. Depth referred to whether information about foods and drinks served was provided, whether ingredients and/or allergens were listed, and whether there was any indication of modifications made for children depending on their age, stage or dietary restrictions. Completeness referred to whether each menu aligned to its corresponding survey responses in relation to the types of meals, snacks, and drinks. This allowed us to determine if certain items, like sugar sweetened beverages, breakfasts, or snacks were being regularly communicated to parents via the menus. Clarity referred to whether foods listed on the menus were objectively clearly described and understandable (such as, ‘vegetables served’ versus ‘broccoli, peas and sweetcorn’) categorized as ‘yes’, ‘mostly’, ‘somewhat’, and ‘no’. A justification for each clarity assessment was recorded by each author. Findings were written up narratively based on the study’s objectives.

### Ethical considerations

Ethical approval was obtained before the study commenced from the London School of Hygiene & Tropical Medicine Ethics Committee (ref: 22664).

## RESULTS

### Participants

Three hundred and twenty-two (322) childcare settings completed the survey, 56 of whom also submitted their menus. Of those, five were excluded because the attachment was either not a menu or the image quality was too poor to read. Thus here we report on 51 menus. For more information on the results from the survey, please see (Warren *et al*. 2024). Details about the settings that submitted menus are provided in Table 1.

**Table 1.** Description of settings that submitted menus.

Regions	Number	Unweighted %	Weighted %
North (NE, NW, Y&H)	12	23.5	24.1
Midlands (EM, WM, London)	15	29.4	30
South (SE,S W, London)	24	47.1	45.9
Nursery type			
Local Authority nursery	2	3.9	3.9
School-based nursery	1	2	2
Private nursery	40	78.4	77.9
Voluntary/Community/Charity nursery	8	15.7	16.2
Deprivation			
Deprived	13	25.5	26.1
Average	8	15.7	14.3
Not deprived	30	58.8	59.6
Nursery ownership			
Independent nursery (sole trader)	37	72.6	72.3
Small chain	10	19.6	19.7
Large chain	1	2	2
No response	3	5.9	6.1
Length of daily provision			
Sessional day care (2–3 hours)	3	5.9	6.1
Extended day care provision (6–8 hours)	6	11.8	12.2
Full-day care nursery (>8 hours)	42	82.4	81.8

NE (North East), NW (North West), Y&H (Yorkshire and the Humber), EW (East Midlands), WM (West Midlands), SE (South East), SW (South West)

Further analysis (found in Supplement S2) shows that the settings that submitted menus are similar to those that completed the survey.

### Depth: what do menus communicate about foods and drinks served in nurseries?

Of the 51 survey respondents that submitted readable menus, 15 menus did not describe one or more snack(s) a day, four did not describe breakfast, six did not describe neither breakfast nor snacks, and two settings only described lunch on their menu despite their survey responses indicating that they serve meals and/or snacks throughout the day. Only three provided information about all or nearly all of the ingredients used in dishes. Of those three settings that reported all or nearly all ingredients, all reported using guidelines: two reported using the Example Menus for Early Years Settings in England and the other setting reported using EBSB guidelines in their survey responses. Two of those three settings also highlighted common allergens, an uncommon but important food-safety practice.

Just over half (27/51) of menus did not provide information about how meals are changed for children with dietary restrictions. Of the 24 remaining settings, some information was provided but the depth varied: 13 listed specific vegetarian alternatives, two indicated that vegetarian options are available but did not say what those would be, four had a generic disclaimer that restrictions would be catered for, and two menus indicated that meat, dairy, and lentil-free alternatives were available. One menu went beyond all others regarding the depth of information. The menu was stratified by ‘unrestricted’, ‘vegetarian’, ‘highly restricted’, ‘weaning lunch’, and ‘babies finger food’ options, not only catering for specific ethical, religious, and allergy-based restrictions but also those based on children’s ages and development stage. The specification of how foods would be prepared differently depending on children’s age and developmental stage was rare, with only five settings providing this information. One menu provided a QR code and website for parents/carers to access detailed allergen and nutrition information.

### Completeness: how does a menu align with the corresponding setting’s survey responses?

Of the 51 analysed menus, 19 corresponded to the submitted survey responses regarding which meals and snacks they serve on a daily basis. According to the survey responses, 75% (38/51) of settings serve breakfast. However of those, 34% (13/38) do not include information about breakfast on their menus. Just under half (47% [24/51]) of childcare settings report serving breakfast on their menus. Of the settings that reported serving breakfast, 75% (18/24) listed the same breakfast every day and 25% varied their offering (6/24). It was unclear on one menu whether the first items listed were breakfast foods or early morning snack foods. Of the 36 settings that reported serving snacks, the vast majority (71% [25/36]) described them clearly. Four menus did not include information about snacks, and seven menus were vague, making it difficult to assess what a child may have been offered at that time. Examples of descriptions which were felt to be vague included ‘sandwiches’ or listed a wide variety of fruits, vegetables, proteins, and carbohydrates that may be available.

Information about beverages was absent from most menus (69% [35/51]). Three settings that did not provide any information about beverages on their menus reported in the survey that they served flavoured milks and/or squash (also called cordial or dilute; a concentrated fruit-flavoured syrup diluted with water to make a drink), both of which are discouraged in the guidance documents. Seven settings provided some information, often related to beverages offered during meals *or* snacks, but not both. Only nine of the 51 menus had clear and consistent information about beverages across the day.

### Clarity: are foods listed on the menus clearly described and understandable?

**Figure 1.**
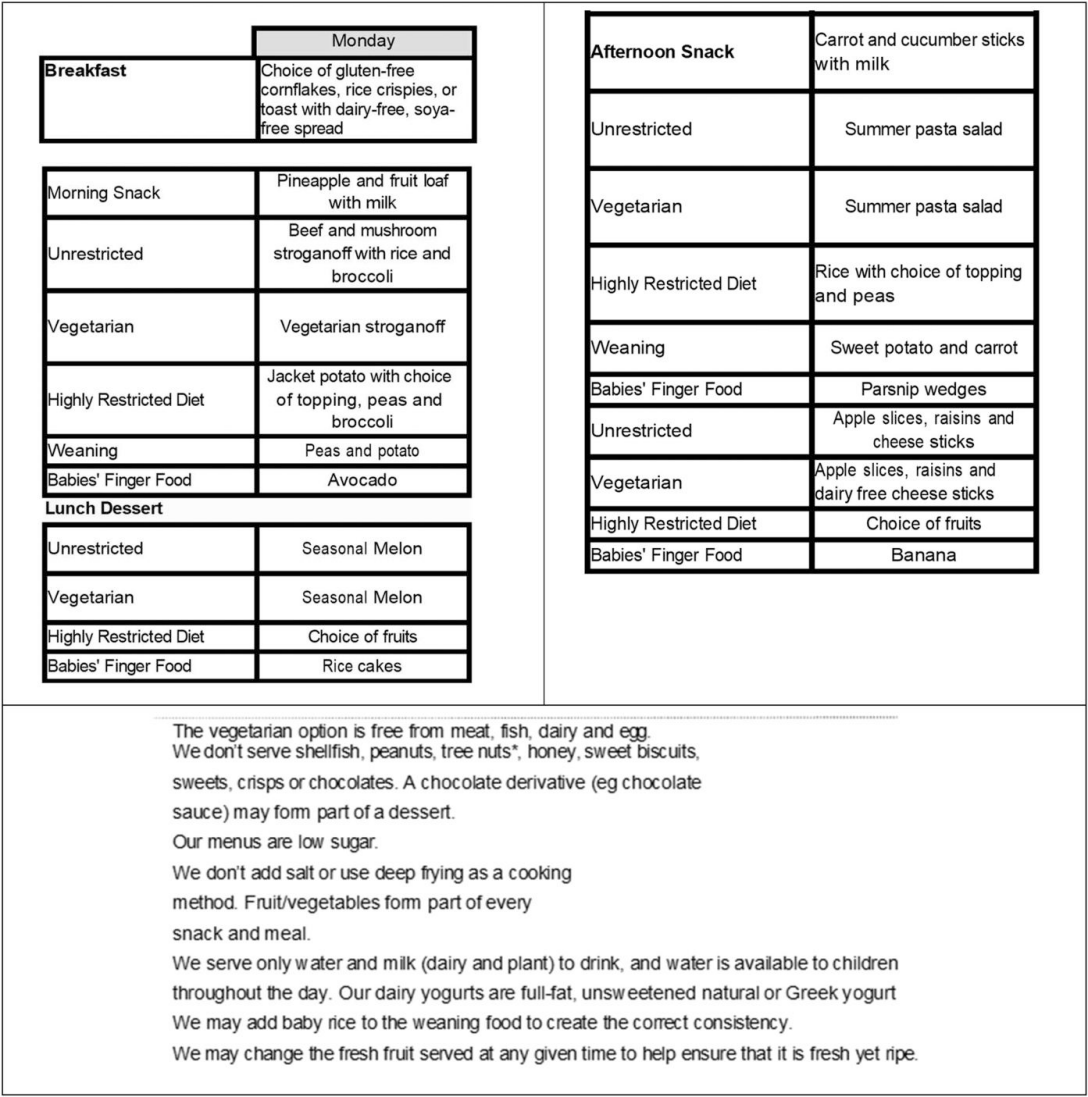
Example of a clear and detailed menu.

**Figure 2.**
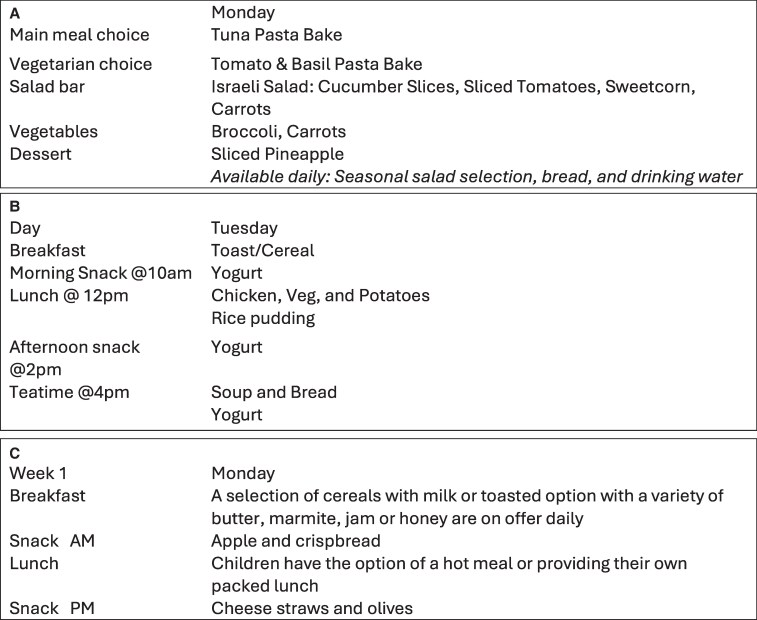
(a, b, c) Examples of menus of limited clarity.

Nineteen of the 51 menus were assessed as being clear (see Fig. 1 e.g.). However the majority of menus were judged to have provided ‘some’ but not enough detail, as illustrated in Fig. 2a, b and c. Key deficit areas included generic descriptors of food such as ‘soup’, ‘fruit and vegetables’, ‘sandwiches’, and/or ‘wraps’ (7/51 settings). Ten settings named dishes but did not provide parents with information about what is in those dishes, e.g. ‘Israeli salad’, ‘Mediterranean chicken stew’, or ‘Japanese jiggly cake’. In three settings, dishes were provided with child-friendly or exciting names but were not informative for carers. Examples include ‘magic’ and ‘pirate’ pasta. Moreover, while some dishes, such as ‘toad in the hole’ may be understood by carers from the UK, they may be unclear to people from other countries. Five settings were determined to provide insufficient details on what was being served during meal and snack time.

Key limitations included rare or occasional dishes that may not be understood such as ‘cowboy salad’, ‘Aussie pie’, or ‘Tex-Mex chilli with rice’; whether the chilli contained beef, vegetables, and/or beans-all plausible options was not specified.

Clear menus also offered creative ways of communicating information. For example, one nursery provided a cumulative daily and weekly count of the number of distinct fruits and vegetables offered to children, and another colour coded foods so that carers could more easily see the distribution of fruits, vegetables, protein, carbohydrates, and dairy items offered to their child. Another made a conscientious effort to improve children’s’ dietary diversity and appreciation for foods from different regions by serving different flavours and types of stir-fries, curries, lasagnes, and soups. One provided carers with a QR code to access more detailed information about ingredients and nutritional information. Finally, one nursery sought to reduce waste by serving left over snacks from earlier in the week every Friday.

## DISCUSSION

This research is the first to our knowledge to describe in detail the quality and depth of information provided to carers by nurseries in England. Our analysis echoes research conducted by Fix Our Food and Bremner & Co in their Yorkshire-based case study which also found a wide variety in the depth of information contained in menus (Brackley *et al*. 2024). For carers, information in menus is important both when choosing a childcare setting and, on a more regular basis, when carers may be trying to provide a balanced diet for their children. In order to be most helpful to carers, menus could be distributed early so that family and setting-based meals can complement each other in terms of nutrition and dietary diversity. As substantiated in a 2022 longitudinal analysis of factors that influence parents’ provision of food for infants and young in England, many mothers in the study reported relying on childcare providers to offer nutritious meals to their children, removing the pressure to provide a substantial meal in the evening (Coleman *et al*. 2022). This is pivotal because decisions on what to serve children in the evening is influenced by what parents think their children have eaten during the day, and they menus are one tool that informs those decisions. Menus are also important for settings as a tool to ensure compliance with the EYFS framework.

The fact that 85% respondents did not share a menu could be due to a number of barriers including but not limited to not having a menu, technical difficulties, or time constraints. Earlier qualitative work by Warren *et al*. showed that staff in the early years sector in England reported serving multiple roles, including education and nutrition provision as well as vital family and social support (Warren *et al*. 2022). In a complex context of various providers types and iniquitous provision of free or low-cost trainings, award schemes, and menu and food-related policy reviews (Warren *et al*. 2022), disparities in the quality of menus, even when focusing only on nurseries, was apparent. Moreover, while childcare settings are trying to keep pace with a rapidly changing policy landscape, menus may not be a top priority area for staff.

Having clear and easy to understand menus is not just about convenience or curiosity; transparency about food is also about safety, particularly for children with allergies. Of the 51 menus included in this analysis, only two included information about common allergens. While much of the literature in this area has been on the importance of establishing a healthy diet in early life and compliance with national guidance, food safety-including allergies and intolerances- is also important for parents, care providers, and regulators.

Earlier research with 59 parents about their involvement in childcare settings food provision found that parents were concerned about menus lacking variety (Williams *et al*. 2022). Menus are an important opportunity for childcare settings to communicate with parents and carers, given the importance of a young child’s diet and development related to food, and in light of the growing understanding of the importance of the parent-practitioner partnership in childcare settings (Kambouri-Danos *et al*. 2017). This is especially relevant in a context where the information contained in menus did not correspond to the information nursery staff provided in their survey, where settings reported serving drinks like soda pop, squash, and flavoured milk. Potential discrepancies between menus and served foods should be explored in future studies in the UK. In the USA, evidence about discrepancies between menus and foods served varies widely: one cross-sectional study found 94%–100% agreement, (Dave and Cullen 2018) while a separate cross-sectional study of Head Start centres found only four meals of 269 (1.5%) matched their menu description. (Fleischhacker *et al*. 2006) It should be noted however that this study was only carried out in one setting, so no attempts at generalizability should be attempted.

Creative or innovative practices were seen in some of the menus, providing insights into some ways that nurseries are improving menus on their own volition. Fruit and vegetable tallies may show parents how easily dietary diversity can be increased, while serving children left-over snacks on Fridays may be a way to open up discussions with children and parents about food the importance of reducing food waste.

### Practice considerations

Findings from this analysis show a number of practical ways that childcare settings can improve their menu-related practices. Firstly, menus can be communicated clearly and in multiple ways with parents so that the foods served at home and in the setting complement each other, ensuring nutritional balance. Menus should including greater details, paying particular attention to breakfasts, snacks, and beverages. Moreover, these menus should more accurately reflect the foods and drinks that are actually being offered to children. Greater transparency about how foods are served based on children’s age and stage, as well as modifications based on restrictions and allergies would also benefit parents and demonstrate more thoughtful engagement with the EYFS requirements. Finally, in a sector that is already stretched, settings should be able to access free training and support in developing and communicating menus, as well as compensation for the time required for staff to upskill.

### Wider policy considerations

These findings are only useful if contextualized in the wider context, both in terms of policy and in challenges faced by childcare settings. Childcare in England is expensive (OECD 2020) and childcare settings are chronically underfunded and poorly supported by the government (Warren *et al*. 2022). The recent expansion in ‘free’ childcare provided to working parents has led to an increase in the number of children and hours spent in childcare settings (Brewer *et al*. 2022). However, the early years sector has reported that the reimbursement rate paid by the government does not cover their costs (Warren *et al*. 2022) Moreover, ‘consumables’, including food and diapers, are not covered by the subsidy, forcing childcare settings to employ multiple strategies to manage cost, including increasing charges for parents not using the scheme (Early Years Alliance and LEYF Nurseries 2022). In deprived areas this has been described as a ‘double whammy’ where childcare settings provide food for children who attend nursery for the first time because they became eligible for free care and who also do not have many parents earning enough to cross-subsidize the costs the government does not pay for (Warren *et al*. 2022).

Stakeholders from the early years sector report being overworked, under-valued, insufficiently funded (Early Years Alliance 2021), and pressurized to fill an increasing number of roles for families, all the while responsible for feeding children attending childcare settings. Thus while childcare settings can play a crucial role in the health promotion of young children during a time of vital development, the wider policy context and challenges faced by childcare settings and families must be addressed. Insufficient attention has been paid to the upstream policies and the social and economic determinants of health that enable or constrain settings’ ability to implement effective interventions and encourage healthy diets during a crucial developmental stage, such as increasing access to free early years meals, and encouraging the Healthy Start programme (UK Government 2025a, b) and extending it to all pre-school children (Bryant *et al*. 2025). Greater policy prioritization is required, and this should be done through a dedicated ministerial lead for early years food and health. Financial support and training are essential via the allocation of protected food funding for the early years sector (Bryant *et al*. 2025). This will ensure that settings can provide a healthy food environment, supported by clear and detailed menus. A higher minimum level of support should be available across the early years sector to better serve children from families with the greatest unmet social and economic needs. These concerns are reflected in the authors’ earlier research on the early years sector in England (Warren *et al*. 2022).

### Limitations

This analysis of childcare settings menus in England finds that first, few surveyed settings (51 of 332, or 15%) returned a legible menu. Although settings were not required to upload a menu to complete the survey, it is likely that those without menus, or with menus they believe do not meet industry or voluntary standards, were less inclined to submit them for analysis than those confident in the quality of their provision. The menus we analysed are therefore likely to be healthier than the sector overall. As such, the need to better guidance and greater support is likely to be more urgent than is evident from this study.

Menus are only one information source and staff may report more detailed information about food consumption to parents at pick-up or via apps, as was documented in our related research on parental involvement in childcare settings’ food decisions and practices. (Williams *et al*. 2022) This may be particularly relevant for breakfasts and beverages, which were commonly missing from menus. Moreover, menu assessments are unable to account for the fact that children may eat multiple portions, some, or none of the food on offer during a given meal or day. This analysis is not intended to suggest that menus serve as a way to assess what is eaten, only what is on offer. In both our findings that some settings serve flavoured milk and/or squash, as well as research from the US indicate the food reported on menus is healthier that what is served; although the US-based research did not find any significant differences between the dietary quality of what was served than consumed. (Kroeger *et al*. 2020) Future research should include more detailed descriptions of what is served and eaten in settings, and how wasted food can be minimised.

Our original research plan included analysing settings’ compliance with nutritional guidelines, but given how little information the menus contained, this proposal proved unfeasible and the study was modified to explore the depth of information contained in the menus.

## CONCLUSIONS

Childcare settings can be crucial sites of health promotion for infants and young children through the provision of healthy food. Inconsistencies in the menus provided is symptomatic of the entrenched challenges faced by childcare settings. Improving the upstream policies and the social and economic determinants of health is essential to enable settings to provide a consistent, good quality and health promoting food environment.

## Supplementary Material

daaf179_Supplementary_Data

## Data Availability

The data supporting the findings of this study are not publicly available as consent for analysis conducted outside the study team was not explicitly sought or approved from the London School of Hygiene & Tropical Medicine’s Ethics Committee (ref: 22664).
