# Supplementary material for: Are childcare settings’ food menus fit for purpose? A qualitative analysis in England
Source: Health Promot Int. 2025 Oct 30;40(6):daaf179. doi: 10.1093/heapro/daaf179 (PMC12574674; doi:10.1093/heapro/daaf179)
Supplement: daaf179_Supplementary_Data [file daaf179_supplementary_data.zip › Supplement 1_Data extraction sheet .docx]

**Supplement 1: Data extraction columns**

Is there alignment between their menu and survey response?

If no, explain your answer

If breakfast is served, is it the same every day?

Are all snacks named?

If no, explain your answer

Is information about beverages on menu?

What information about beverage is given?

Are meal or snack components listed?

If no, explain your answer

Is every ingredient listed? (e.g. what is in cheddar cheese? What is in rice crackers?)

Example

Is information provided about alternative dishes for children with dietary restrictions ?

Are allergens listed?

If so, which ones and how?

Is there anything to indicate that the menu changes throughout the year?

What?

Are adjectives used to describe the food? (e.g. creamy, hearty, succulent)

Does the menu offer other relevant information?

If so, what?

Is there evidence of any other innovative practice on the menu?

If so, what?

Does the menu state how it will be modified for different ages and stages of children in setting?

If so, what

Are vegetables ever described as hidden?

Other final comments on information contained in menu
