# Supplementary material for: Are childcare settings’ food menus fit for purpose? A qualitative analysis in England
Source: Health Promot Int. 2025 Oct 30;40(6):daaf179. doi: 10.1093/heapro/daaf179 (PMC12574674; doi:10.1093/heapro/daaf179)
Supplement: daaf179_Supplementary_Data [file daaf179_supplementary_data.zip › Supplement 2.docx]

Supplemental file 1:

| **Regional distribution of nursery settings** | | |  |
| --- | --- | --- | --- |
| Region | **Number** | **Unweighted %** | **Weighted %** |
| North(NE, NW, Y&H) | 79 | 24.5 | 24.5 |
| Midlands(EM, WM) | 94 | 29.2 | 29.2 |
| South(SE, SW, London) | 149 | 46.3 | 46.4 |
| Total | 322 | 100 | 100 |

* NE (North East), NW (North West), Y&H (Yorkshire & the Humber), EW (East Midlands), WM (West Midlands), SE (South East), SW (South West)
